# Simulating the Use of Discontinuous Patterned Hydrogel to Improve Inter‐Electrode Resistance on Electrode Arrays

**DOI:** 10.1111/aor.15030

**Published:** 2025-06-08

**Authors:** Mark L. Reeves, T. Jamie Healey, Avril D. McCarthy

**Affiliations:** ^1^ Clinical Engineering, Royal Hallamshire Hospital Sheffield Teaching Hospitals NHS Foundation Trust Sheffield UK; ^2^ NIHR Long Term Conditions HealthTech Research Centre – Devices for Dignity, Royal Hallamshire Hospital Sheffield Teaching Hospitals NHS Foundation Trust Sheffield UK

**Keywords:** electrical stimulation, electrode array, inter‐electrode resistance, patterned hydrogel, stimulation current density

## Abstract

**Background:**

A novel form of sensory stimulation aiming to treat spasticity has been developed, and a clinical trial is currently underway. This uses an electrode array controlled by a programmable 64‐channel stimulator to spatially vary the electrical stimulation over time. However, when a continuous layer of hydrogel interfaces between the array and skin, stimulation spreads, causing lower current densities applied over larger areas of tissue. A new approach was developed, modeled, and tested, utilizing discontinuous patterned hydrogel to improve inter‐electrode resistance on electrode arrays.

**Methods:**

Finite‐difference modeling was used to estimate stimulation distribution within the hydrogel and subcutaneous tissue under the electrode array. Repeated simulations modeled changes due to variations in hydrogel, skin, and subcutaneous tissue resistivity. Properties of both continuous sheets and patterned hydrogel were used for the simulation. Physical prototypes of the continuous and patterned hydrogel were manufactured and tested for comparison with the simulation.

**Results:**

Simulation results showed a reduced spread of stimulation between electrodes when using the discontinuous patterned hydrogel compared to the continuous hydrogel. This was demonstrated consistently for all variations in hydrogel, skin, and subcutaneous tissue resistivity. Laboratory testing supported the simulation results and suggested the improved performance of the patterned hydrogel, compared with the continuous hydrogel, may become more substantial over time.

**Conclusions:**

While the simulation only approximates the stimulation distribution on electrode arrays, the results do show potential benefits of utilizing discontinuous patterned hydrogel to increase inter‐electrode resistance. Laboratory testing and initial feedback from the clinical trial support the results indicated in the simulations.

## Graphical abstract

Stimulation spreads across electrode arrays using continuous hydrogel for the electrode‐skin interface, causing lower current densities over larger areas. A new approach was developed, modeled, and tested, utilizing discontinuous patterned hydrogel to improve inter‐electrode resistance on electrode arrays. Simulation and laboratory testing showed stimulation spread reduced when using patterned hydrogel.

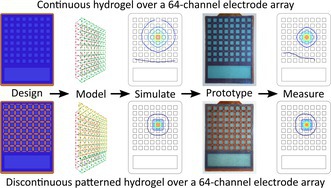

## 1 Background

**FIGURE 1:**
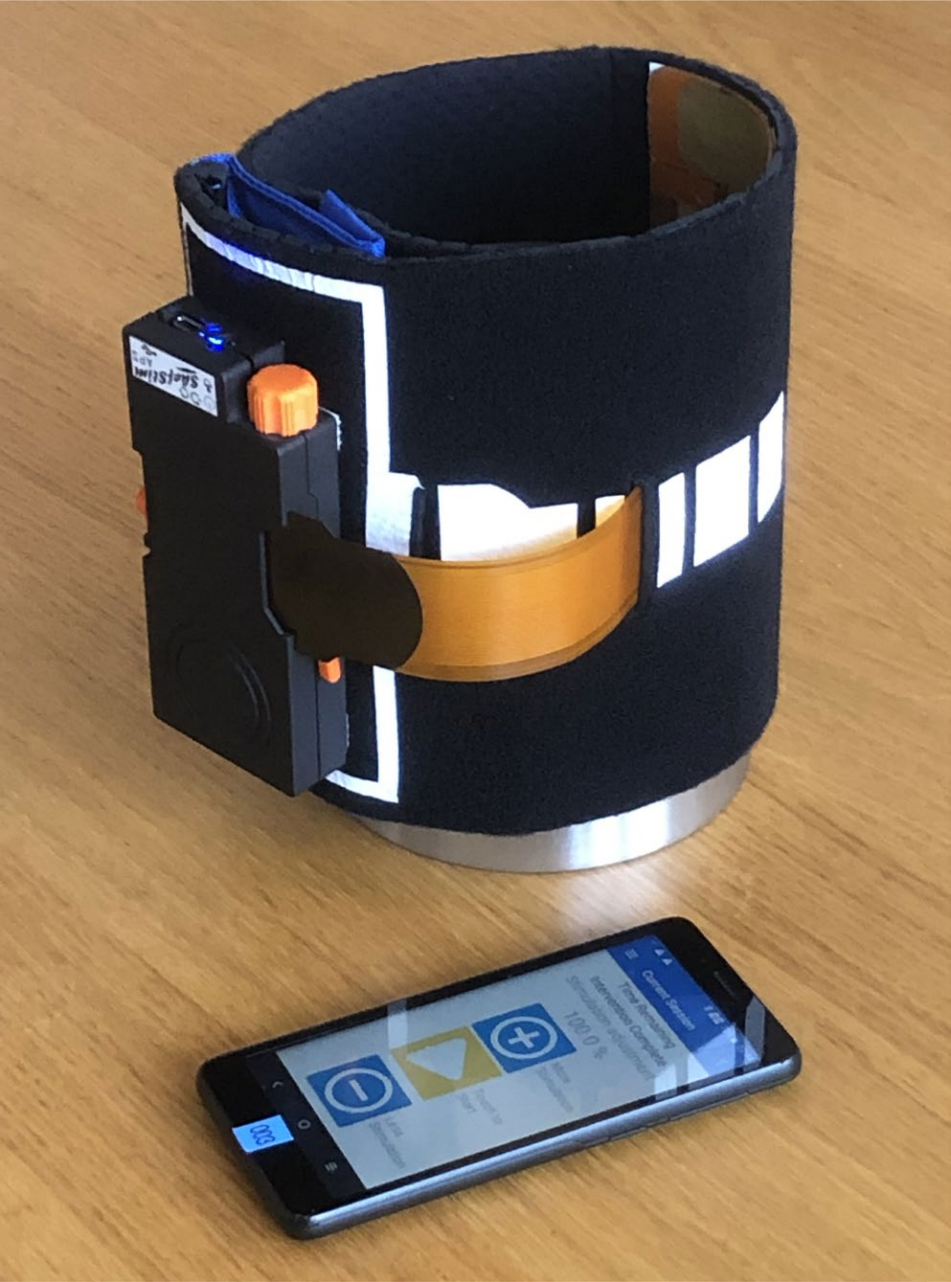
ShefStim APS stimulator connected to the electrode array and attached to the arm sleeve. A smartphone app is used by the clinician for the initial setup of the stimulator and by the participant as a remote control for the stimulator. [Color figure can be viewed at wileyonlinelibrary.com]

In this paper we explore the use of a discontinuous patterned hydrogel to improve the spatial control and longevity of an electrode array stimulation system. This work relates to a clinical trial of a novel sensory electrical stimulation device to treat the debilitating effects of post‐stroke elbow spasticity [1]. The ShefStim APS device (Sheffield Teaching Hospitals, UK) is intended to be used by patients in their homes, either independently or with informal carer assistance. A randomized control trial (RCT) is currently underway as part of the NIHR funded Sheffield Adaptive Patterned Electrical Stimulation (SHAPES) project [2]. Participants in the intervention groups of the SHAPES study wear an electrode array over the triceps area of the upper arm for 1 h each day over a 6‐week period. The array is held in position using a bespoke arm sleeve, designed to facilitate one‐handed donning and doffing, with the stimulator attached to the outer surface (Figure 1). ShefStim APS has been designed so it can be set up in a blinded manner to deliver either Transcutaneous Electrical Nerve Stimulation (TENS), with stimulation in a fixed location, or SHAPES, where there is spatial and temporal variation in the stimulation pattern. The device trial is being conducted with UK Health Research Authority (HRA) ethical approval (Ref: IRAS 309757) and UK regulatory permissions (Ref: MHRA CI‐2022‐0005).

### 1.1 Electrode Array Stimulation

An electrode array consists of multiple electrodes on a single substrate, often arranged in a grid pattern. These may be used for measuring electrical activity or for delivering electrical stimulation. This application focuses on the latter. Each electrode in the array can be independently controlled when used in conjunction with a bespoke programmable stimulator. Different combinations of electrodes on the array can then be activated selectively. Such programmability enables the stimulation location to be controlled directly by the stimulator with no physical re‐positioning of electrodes required [3]. In this case, the array used is an 8 × 8 grid of 7 × 7 mm electrodes, with inter‐electrode spacing of 4 mm. To benefit from having 64 individually controllable electrodes, the applied stimulation needs to be restricted to the region under each of the electrodes that have been programmed to deliver it.

Hydrogel is used to provide an interface between the electrode array and the skin. However, a continuous layer of low resistivity hydrogel causes stimulation current to spread over the whole electrode array. This causes lower current density in the intended stimulation region and leads to the stimulation being applied over larger areas of tissue where stimulation is not required. Effectively, a loss of focus and magnitude for the stimulation occurs, which in turn reduces the potential benefits of using electrode array technology. This is especially important where one would intend to exploit the programmability of the individual electrodes in the array to provide specific therapeutic characteristics, such as varying the locations and timings of where the stimulation is delivered.

### 1.2 Increasing Inter‐Electrode Resistance

Ideally, an anisotropic electrode‐skin interface would be used to provide a low resistance connection between the electrode array and the patient's skin while maintaining a high resistance between the adjacent electrodes on the array. However, commercially available hydrogels are isotropic, and the stimulation spreads between the electrodes and is therefore applied over a larger area.

Other research investigating electrode resistivity has primarily focused on lowering resistivity between electrodes and skin or internal organs [4, 5, 6, 7, 8, 9] rather than increasing resistivity between individual conductive electrodes to prevent current dispersion over an array of multiple electrodes. Agarwala [4] reviewed a range of novel conductive hydrogels and methods of production including 3D printing and highlighted the “issue of lack of the spatial control with current fabrication techniques” and concluded that the “area of conducting hydrogels is still full of unresolved technological challenges.”

Previous work to limit current spread trialed direct electrode‐skin contact, but this caused inconsistency of electrical contact and, importantly, discomfort for the recipient of the stimulation. Sha [10] investigated the influence of hydrogel impedance on comfort, determining a 28% decrease in discomfort with the use of an electrode with high impedance hydrogel compared with a low impedance one. More recent papers assessing discomfort using the transcutaneous electrical stimulation comfort questionnaire (TESCQ) scale have concluded that hydrogel remains beneficial for comfort compared to dry and textile electrodes [11, 12]. The use of individual hydrogel islands (applied over each electrode) had also been explored. However, in practice this was difficult to manufacture for low volume production and required additional adhesion to prevent the hydrogel islands detaching from the array substrate. Bespoke additive manufacturing methods may be possible for mass production. A continuous sheet of high resistivity hydrogel can be comfortable to use and reduce current spread [13]. However, its resistivity decreases over time with prolonged skin contact. This leads to the hydrogel needing to be replaced frequently and would add significant cost, as multiple pre‐prepared arrays with hydrogel sheets in situ would need to be provided to a patient. Needing to replace the arrays frequently would add complexity for stroke patients with hemiplegia.

**FIGURE 2:**
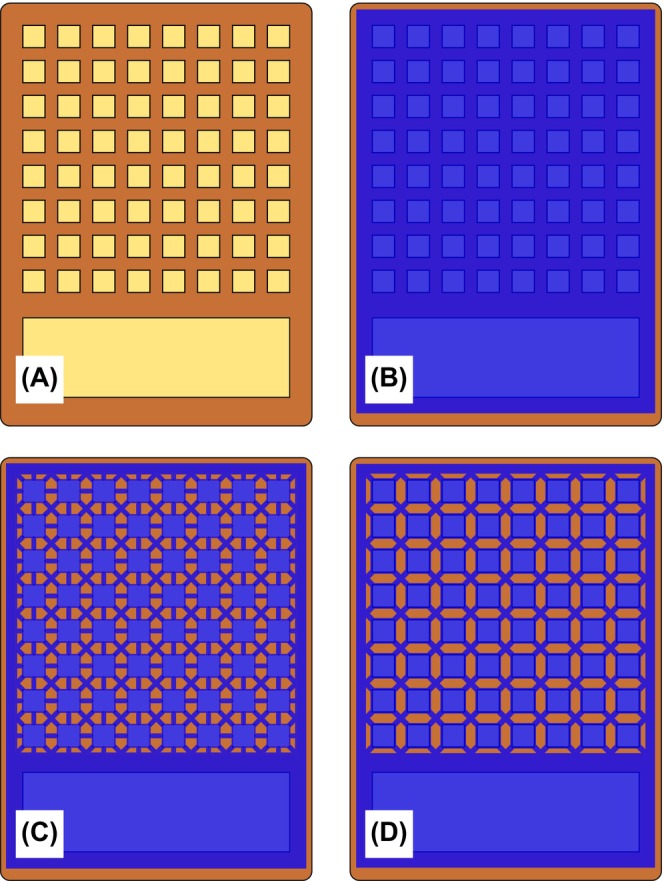
(A) Outline of the electrode array with 64 independently controlled cathodes and a large common anode, overlaid with continuous hydrogel (B), and two alternative discontinuous patterned hydrogel designs: vertical, diagonal, and horizontal supports (C), diagonal supports only (D). [Color figure can be viewed at wileyonlinelibrary.com]

The use of discontinuous patterned hydrogel is a potential solution to these issues. This technique utilizes a sheet of hydrogel covering the entire array, but the discontinuous pattern, created using a subtractive method, introduces air gaps between individual electrodes (Figure 2). Therefore, low resistance can be maintained within the hydrogel between the array electrodes and the skin, while also increasing the inter‐electrode resistance. As the hydrogel is still applied over the electrode array as a single sheet, it is also relatively simple to align with and apply to the array and is mechanically stable.

## 2 Methods

### 2.1 Discontinuous Patterned Hydrogel Design

A number of different design concepts were initially explored for the patterned hydrogel. The inter‐electrode resistance was increased by the perforation of the hydrogel and hence, added air gaps between the areas of hydrogel over each electrode. However, to maintain the structural integrity of the patterned hydrogel and to ensure the electrode arrays were practical to manufacture, inter‐connections were required between the areas of hydrogel.

While the thickness of the hydrogel was predetermined, increasing the length and decreasing the total cross‐sectional area of the inter‐connecting supports between electrodes increased inter‐electrode resistance. However, this also weakened the physical structure of the patterned hydrogel, which needed to overcome the adhesion forces occurring during the removal of the hydrogel from the skin. Using a combination of horizontal, vertical, and diagonal supports (Figure 2C) provided good structural characteristics but increased the total cross‐sectional area and reduced the average length of the supports, hence reducing inter‐electrode resistance. A compromise between the electrical and mechanical factors was achieved by having regions of hydrogel positioned over each electrode that were physically supported by a diagonal lattice pattern (Figure 2D). This design provided appropriate structural support while increasing the inter‐electrode resistance and simplifying manufacturing.

### 2.2 Modeling Stimulation Distribution

Finite difference models were developed to simulate the stimulation distribution under the electrode array while applied to the arm, for both the continuous and discontinuous patterned hydrogel. These models only considered the resistive element of both the hydrogel and tissue impedance, as previous work suggested that the effect of the reactance (the capacitive element) on the current flow was minimal in this application [13].

**FIGURE 3:**
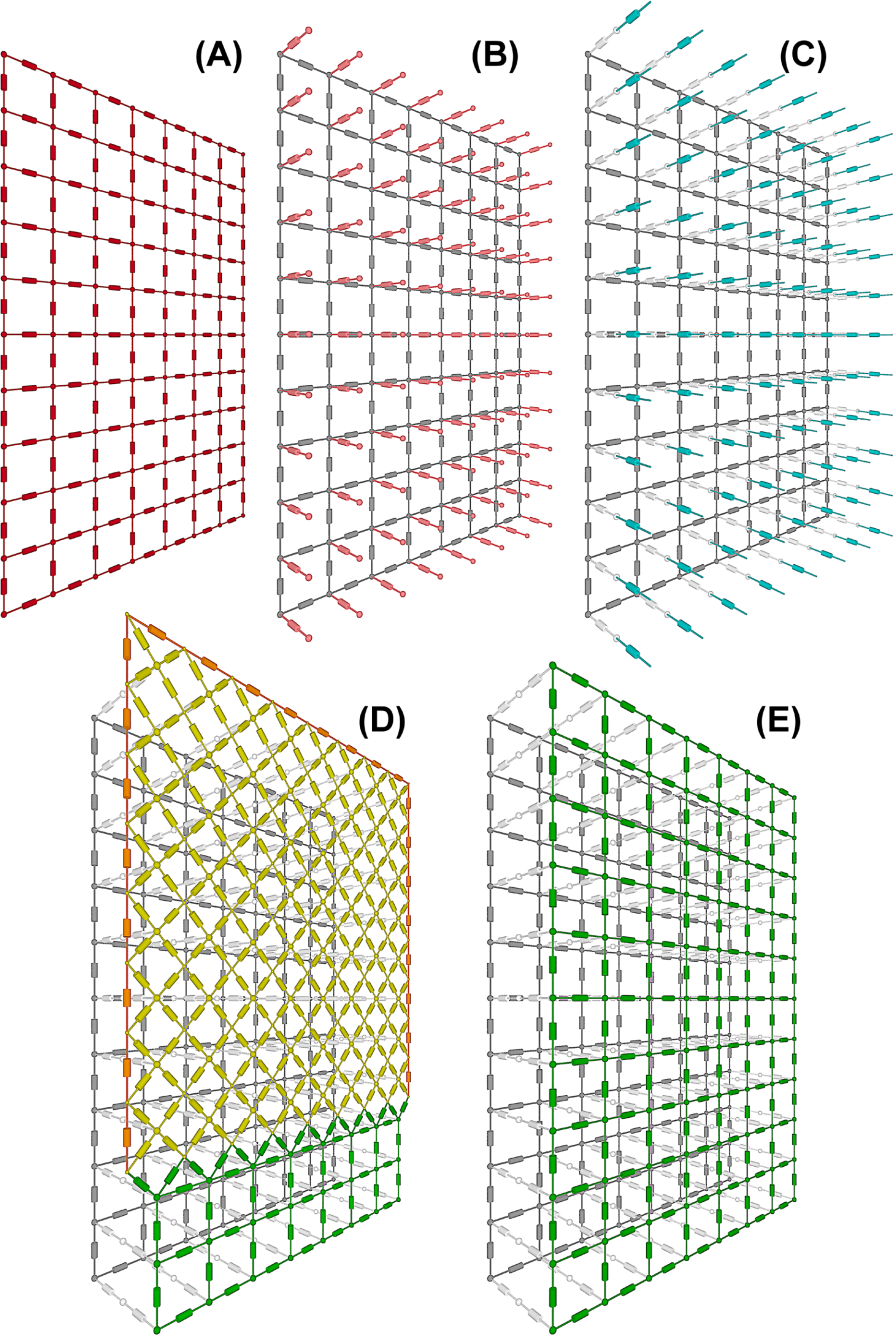
3‐D visualization of the four layers in the simulation models: (A) subcutaneous tissue, (B) skin surface to subcutaneous tissue, (C) electrode to skin surface (hydrogel), and inter‐electrode resistance for (D) patterned and (E) continuous hydrogel models. [Color figure can be viewed at wileyonlinelibrary.com]

While the models were three‐dimensional, for simplicity they were limited to four layers (Figure 3):

1. Subcutaneous tissue resistance (A)
2. Skin surface to subcutaneous tissue resistance (B)
3. Electrode to skin surface resistance of the hydrogel (C)
4. Inter‐electrode resistance within the hydrogel (D and E)

Each layer covered the entire area under the electrode array which included the 64 cathodes, the single large anode, and the gap between the cathode array and the anode. Subcutaneous tissue was modeled as a homogeneous two‐dimensional layer in the form of a simple grid of resistors interconnecting between each node. The resistance directly between two adjacent nodes on the skin surface was assumed to be much greater than the resistance between the skin surface and subcutaneous tissue; therefore, it would have minimal effect on the model and could be excluded. This layer was then simplified down to an array of individual resistors (not interconnected) interfacing between the skin surface and subcutaneous tissue.

Resistance within the hydrogel was separated into two layers, with the electrode to skin surface resistance being modeled as an array of individual resistors in the same configuration as the skin surface to subcutaneous tissue layer. Within the continuous hydrogel, the inter‐electrode resistance was then represented as a simple two‐dimensional grid. However, inter‐electrode resistance in the discontinuous patterned hydrogel required a more complex form of representation. The hydrogel over each cathode on the array was assumed to be at the same potential as the adjacent electrode and could therefore be modeled as a single node. An additional grid of nodes was then added, with each representing the center points within the diagonal lattice structure physically connecting between the areas of hydrogel over each electrode. A continuous strip of hydrogel provided structural support around the outer borders of the array, and this was modeled as resistors between the adjacent nodes on the outer edges of the grid representing the lattice structure. The hydrogel covering the anode was continuous, so this region was simulated as a simple two‐dimensional grid pattern.

### 2.3 Estimating the Range of Resistance Values

The resistivity and thickness of new samples of each grade of hydrogel were measured. Initially, the TG28 grade was found to have a resistivity of 48 Ωm and a thickness of 0.5 mm, while the SRRA grade had a resistivity of 27 Ωm and a thickness of 1 mm. The effect of prolonged skin contact was determined by repeated measurements over a 14 h period of skin application and was found to reduce to approximately 50% of the original values over this time period. In each case (new TG28, new SRRA, used TG28, and used SRRA) the resistor values modeling each different region of the hydrogel were calculated within the simulation, allowing for the length, cross‐sectional area, and resistivity. For the discontinuous patterned hydrogel, the area over each electrode was modeled as 49 mm^2^ and the width of the diagonal lattice structure was 1.4 mm.

Approximate values for skin surface to subcutaneous tissue resistance were measured. A commercially available CE marked stimulator (ODFS Pace, Odstock Medical Ltd., UK) was used to apply the stimulation (180 μs pulse width, 50 mA set current) between adjacent cathodes on the electrode array, each covered with a separate square of hydrogel. The array was applied over the researcher's triceps and the stimulation voltage (applied between adjacent cathodes) and current were measured. The measured current was substantially lower than the set current as the ODFS Pace stimulator is typically used with low resistance electrodes with areas of approximately 25 cm^2^, while the array electrodes have an area of 0.49 cm^2^. Compensating for the known hydrogel resistance and assuming the skin resistance was much greater than the subcutaneous tissue resistance, the skin resistance under each cathode was found to typically have a value of between 10 and 50 kΩ.

The consistency of the subcutaneous tissue in the upper arm was assumed to be primarily either fat or muscle, and typical low frequency conductivity values for each of these tissue types (0.0406 S/m for fat and 0.267 S/m for muscle [14]) were used in separate runs of the simulation.

### 2.4 Implementation of the Model

Scilab, an open‐source numerical computational package (Version 6.1.0, Dassault Systèmes, France), was used to implement the model. Two numeric arrays were created to represent the voltage at each individual node in the model. The first of these arrays stored the values calculated in the previous iteration, while the newly calculated voltages were stored in the second array. Equations were derived to calculate the new voltage at every node relative to the previous voltages at each adjacent connected node and the resistance between the nodes. After these calculations had been completed for every node in the model, the values in the newly calculated array were transferred to the array of previous values, and the whole process was repeated.

A single electrode array node, representing one active cathode, was fixed at a constant stimulation voltage and all nodes representing the anode were fixed at zero. The simulation then went through an iteration process until the sum of all the calculated current values between the electrode array and sub‐cutaneous tissue approached zero. This represented the stable state where the total current flow from the stimulator to the tissue equaled the total current flow from the tissue back to the stimulator.

Two separate models were created, one to simulate the continuous hydrogel, and the second for the discontinuous patterned hydrogel. To investigate the potential range of stimulation spread under different conditions, the simulation for each hydrogel model was repeated for all eight combinations of the maximum and minimum resistivity values for the hydrogel, skin, and subcutaneous tissue.

Initially a range of different hydrogel products, with varying thickness and resistivity values, were considered (ST‐GEL grades: SCBZAB, SRRA, TG24, and TG28, from Sekisui Kasei Co. Ltd., Japan). However, only the TG28 and SRRA grades were still commercially available at the time of testing, and the properties of both of these hydrogels were used for the simulations.

### 2.5 Laboratory Testing

**FIGURE 4:**
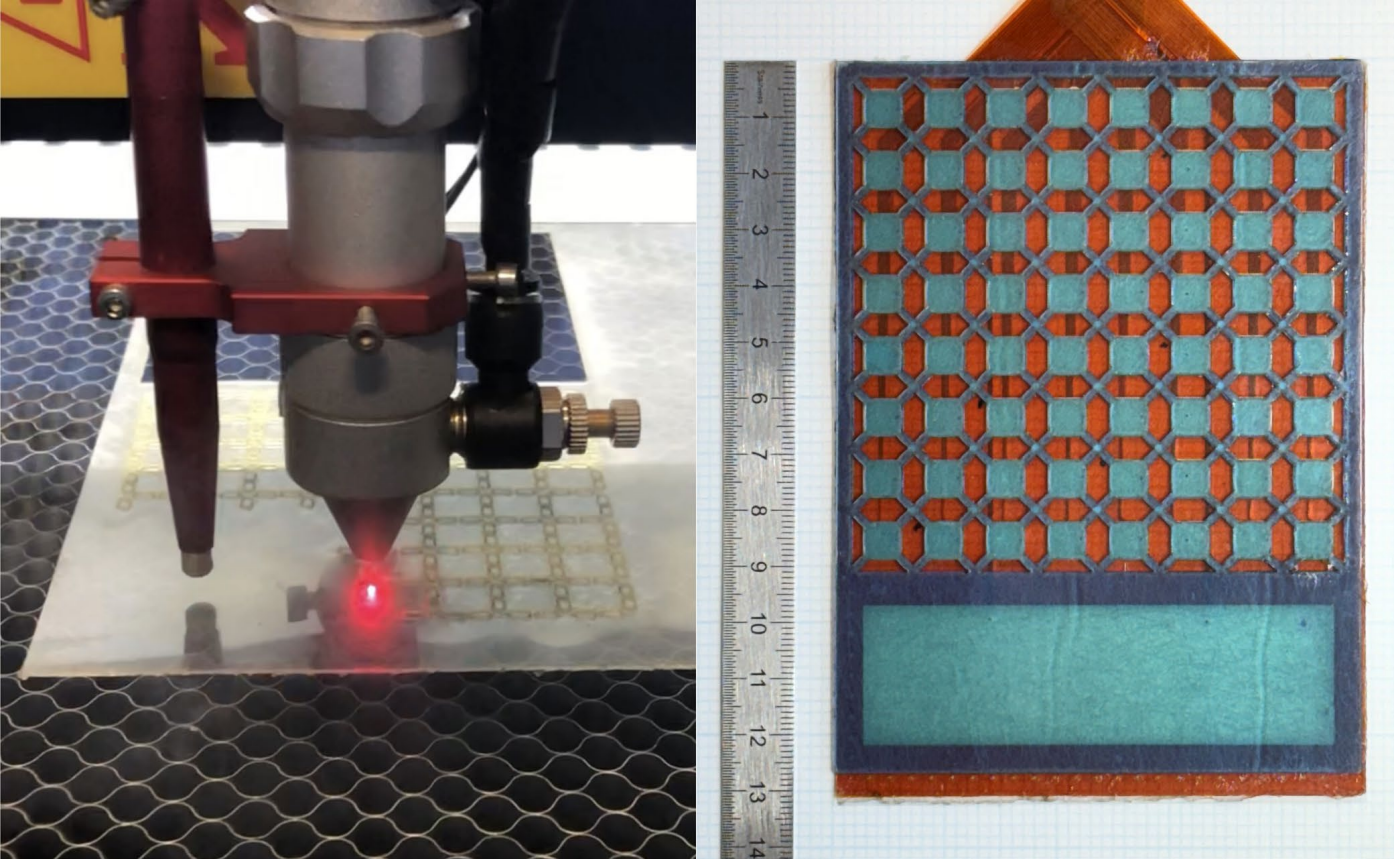
Laser cutting of the hydrogel (left). Laser cut patterned hydrogel applied over a 64‐channel electrode array (right). [Color figure can be viewed at wileyonlinelibrary.com]

Physical prototypes of the discontinuous patterned hydrogel design were manufactured using a CO_2_ laser cutter (TS4060 60 W, Technology Supplies Ltd., UK). The dimensions of the hydrogel pattern were selected so that it could be applied to electrode arrays which had been manufactured for a previous feasibility study [15]. The laser cut hydrogel was manually applied to the electrode array and aligned to fully cover all the electrodes, therefore ensuring they would not come into direct contact with the skin during use (Figure 4).

**FIGURE 5:**
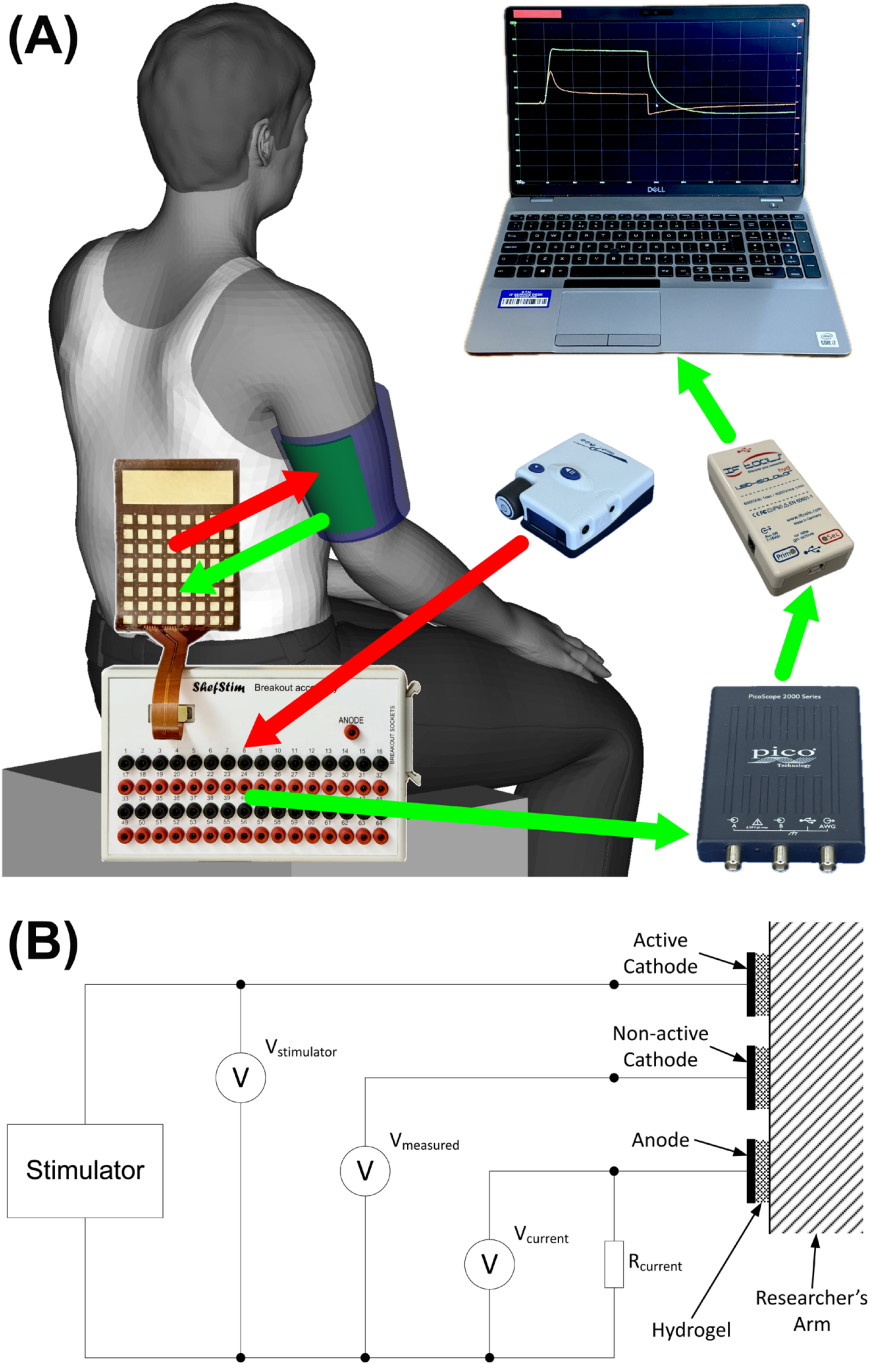
(A) Overview of system used to measure electrode array voltage distribution, showing the stimulation pathway (red) and measurement pathway (green). (B) Circuit diagram of the test system. [Color figure can be viewed at wileyonlinelibrary.com]

The array was applied over the researcher's triceps and then connected to a breakout board. This enabled stimulation to be applied between any combination of two individual electrodes on the array, while simultaneously being able to measure the voltages on surrounding electrodes. An ODFS Pace device was used to apply the stimulation (180 μs pulse width, 50 mA set current) to the equivalent cathode on the electrode array as the one modeled as being active in the simulation. A resistor (with a value significantly lower than the combined resistance of the hydrogel, skin, and subcutaneous tissue) was placed in series between the large anode electrode on the array and the anode connection on the stimulator, to monitor the total current flow. The applied voltage, total current, and voltage on all other cathodes on the electrode array were measured using a USB oscilloscope (PicoScope 2204A, Pico Technology Ltd., UK) connected to a PC via an EN‐60601‐1 compliant USB isolator (ISOUSB‐HVD, IF‐Tools GmbH, Germany). An overview of the system is shown in Figure 5.

To establish how the voltage distribution on the array changed with prolonged use, the voltage on each cathode (relative to the common anode) was measured during stimulation at regular intervals for a total application period of 14 h (7 h a day over 2 days). The experiment was initially carried out with a continuous sheet of hydrogel covering the whole electrode array, including the 64 cathodes and a single anode. Testing was then repeated using the laser‐cut discontinuous patterned sheet of hydrogel.

## 3 Results and Discussion

### 3.1 Simulated Stimulation Distribution

Using the simulated voltage on each node in the hydrogel layer and the voltage on the corresponding node in the subcutaneous tissue layer, the current under each cathode was calculated. The distribution of the current modeled in the simulations showed substantial variation with the different combinations of resistivity values for the hydrogel, skin, and subcutaneous tissue (Table 1).

**TABLE 1:** Comparison between the percentage of total current applied directly under the active electrode for the high and low resistivity values of hydrogel, skin, and subcutaneous tissue. Both the continuous and patterned hydrogel simulations were repeated for two grades of hydrogel (TG28 and SRRA).

Resistivity			Percentage of total current under the active cathode			
Hydrogel	Skin	Tissue	Continuous hydrogel		Patterned hydrogel	
			TG28 (%)	SRRA (%)	TG28 (%)	SRRA (%)
High	Low	High	51.7	30.1	87.0	80.0
High	Low	Low	50.3	26.9	86.5	79.0
Low	Low	High	39.2	23.4	78.2	68.6
Low	Low	Low	36.7	19.2	77.2	66.7
High	High	High	23.3	13.3	59.8	47.6
High	High	Low	22.3	12.0	59.1	46.5
Low	High	High	16.9	10.7	45.2	34.3
Low	High	Low	15.5	9.1	44.0	32.7

**FIGURE 6:**
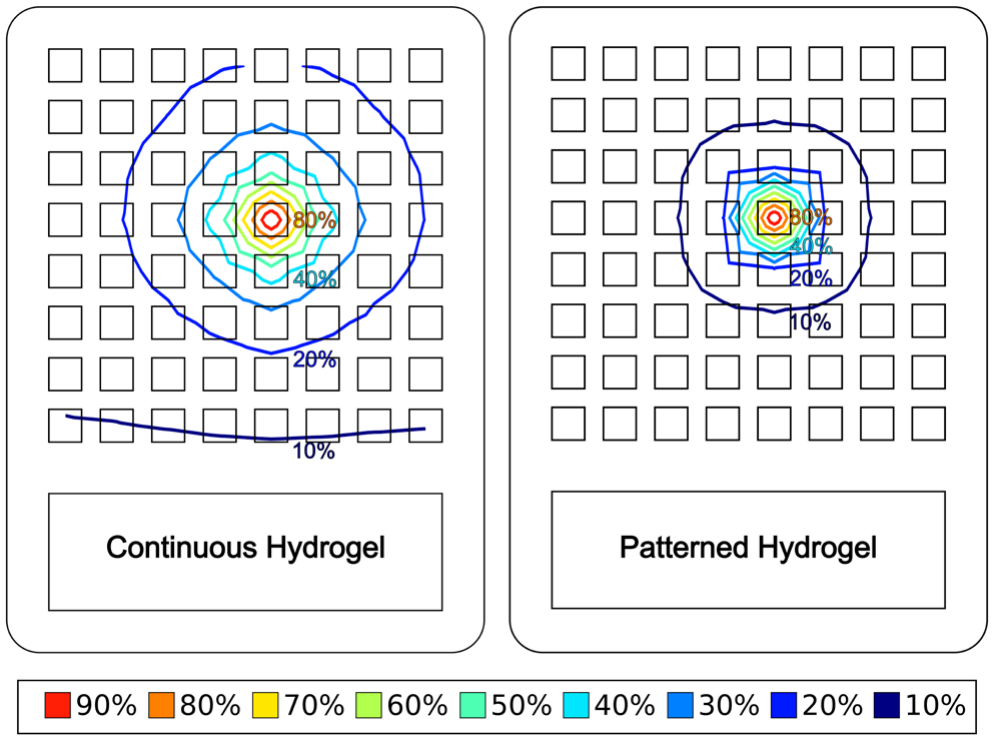
Contour plots (normalized relative to the fixed voltage on the single active cathode) comparing the simulated array voltage distribution (worst‐case conditions) overlaid on the outline of the electrode array with continuous TG28 hydrogel (left) and patterned TG28 hydrogel (right). [Color figure can be viewed at wileyonlinelibrary.com]

The worst‐case scenario was when the maximum spread of stimulation occurred, as this would cause the lowest proportion of stimulation to be applied directly between the active electrode and the adjacent targeted area of subcutaneous tissue. The simulation data suggested that this scenario would occur when the hydrogel and subcutaneous tissue resistivity values were low, and the skin resistance was high. These conditions were found to cause the greatest spread of the stimulation in both the continuous and patterned hydrogel models, and for both the TG28 and SRRA grades of hydrogel. The simulated array voltage distribution under these conditions (superimposed over the outline of the electrode array) is illustrated in Figure 6.

The simulation showed that patterned hydrogel consistently improved the focus of the stimulation in comparison with the equivalent simulation conditions for continuous hydrogel. Even in the worst‐case scenario, the patterned hydrogel still outperformed the continuous hydrogel under most conditions. The relative difference between the percentage of total current under the active electrode in the continuous and the patterned hydrogel was most significant in the worst‐case scenario.

SRRA grade hydrogel was both thicker (decreasing the inter‐electrode resistance while also proportionally increasing the electrode to skin resistance) and had a lower resistivity than the TG28 grade hydrogel. As expected, the simulation demonstrated that these factors caused poorer SRRA performance under all conditions, leading to greater spread of the stimulation over the array. For this reason, the TG28 hydrogel was selected for the laboratory testing.

### 3.2 Measured Stimulation Distribution

**FIGURE 7:**
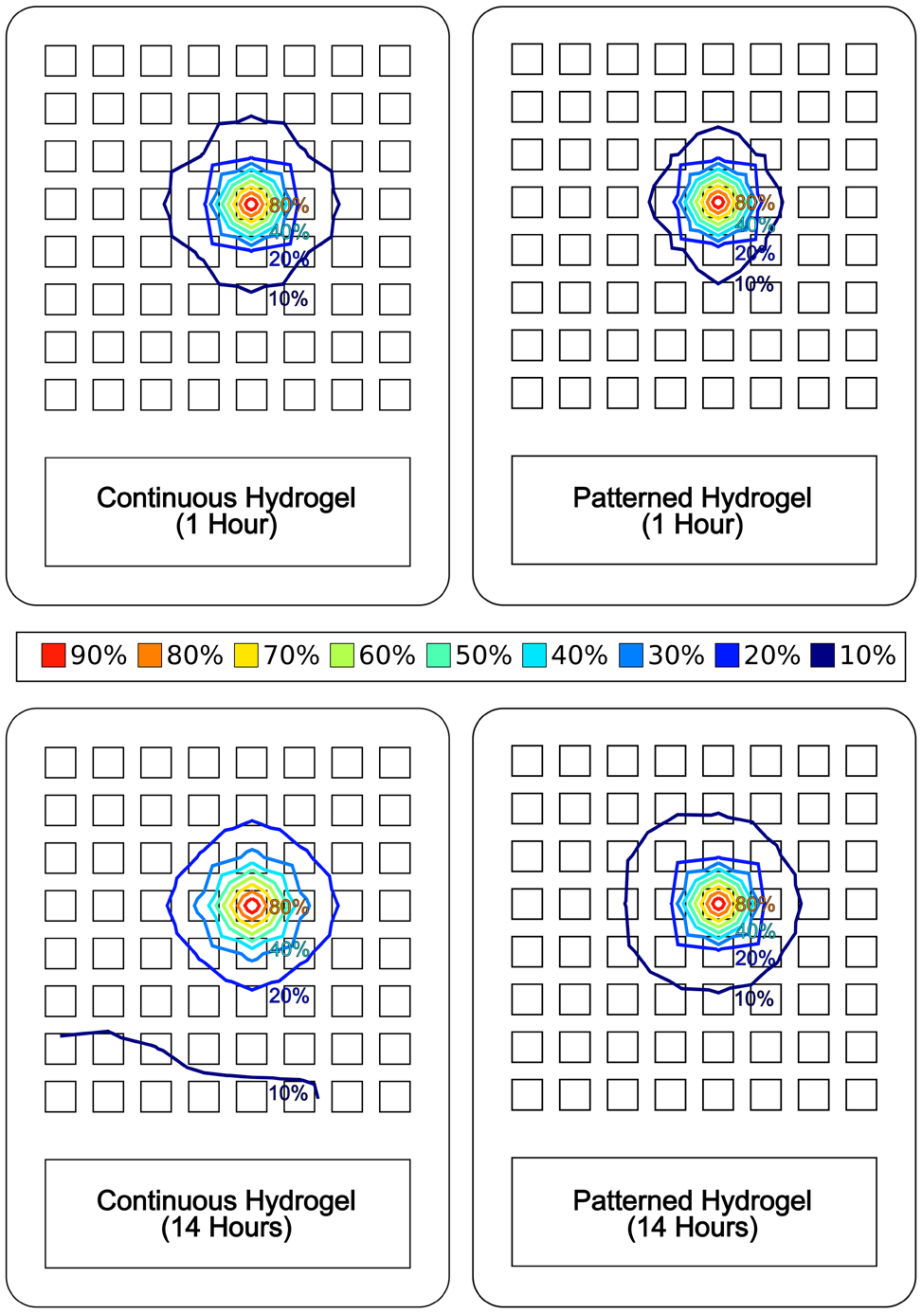
Contour plots (normalized relative to the stimulation voltage applied on the single active cathode) comparing the measured voltage distribution: Continuous hydrogel after 1 h (top left), patterned hydrogel after 1 h (top right), continuous hydrogel after 14 h (bottom left), patterned hydrogel after 14 h (bottom right). [Color figure can be viewed at wileyonlinelibrary.com]

During the laboratory testing, only the applied stimulation voltage, total current, and voltage distribution on the array could be directly measured. The measured voltages from each cathode on the array were recorded after the array had been applied to the researcher's arm for 1 h and again after 14 h (7 h a day for 2 days) of use. The voltages were normalized relative to the stimulation voltage applied to the single active cathode. This data set was imported into Scilab for processing, and a 2‐D contour plot was generated (Figure 7).

Visual inspection showed the laboratory test results were consistent with the simulations and a greater spread of the stimulation was observed with the continuous hydrogel, while there was better retention of focus with the patterned hydrogel. The difference between the normalized voltage distribution on the continuous and patterned hydrogel was relatively subtle after 1 h of application to the arm (continuous hydrogel: 9 electrodes > 10%, patterned hydrogel: 5 electrodes > 10%). However, after 14 h of use, there was a much greater difference (continuous hydrogel: 55 electrodes > 10% and 9 > 20%, patterned hydrogel: 9 electrodes > 10% and only the active electrode > 20%). The changes in the voltage distribution on the array after 14 h of use were consistent with the simulated effects of a 50% reduction in the resistivity of the hydrogel.

### 3.3 Limitations

Current flowing between individual electrodes and the adjacent subcutaneous tissue could not be measured directly. For all the laboratory testing, the electrode array was applied in the same position and orientation on the researcher's left arm (over the triceps). An arm sleeve from the SHAPES clinical trial was used to hold the electrode array in the correct position and to apply a consistent pressure to the array, even after repeated application/removal and changing between the continuous and patterned hydrogel. While the resistivity of the skin and subcutaneous tissue could not be directly measured, in these circumstances it was reasonable to assume that they did not vary significantly during these experiments. Therefore, the spatial variation in the current distribution would be similar to the voltage distribution across the array. The voltage distribution contour plots (with the continuous and patterned hydrogel, after 1 and 14 h) are directly comparable.

**FIGURE 8:**
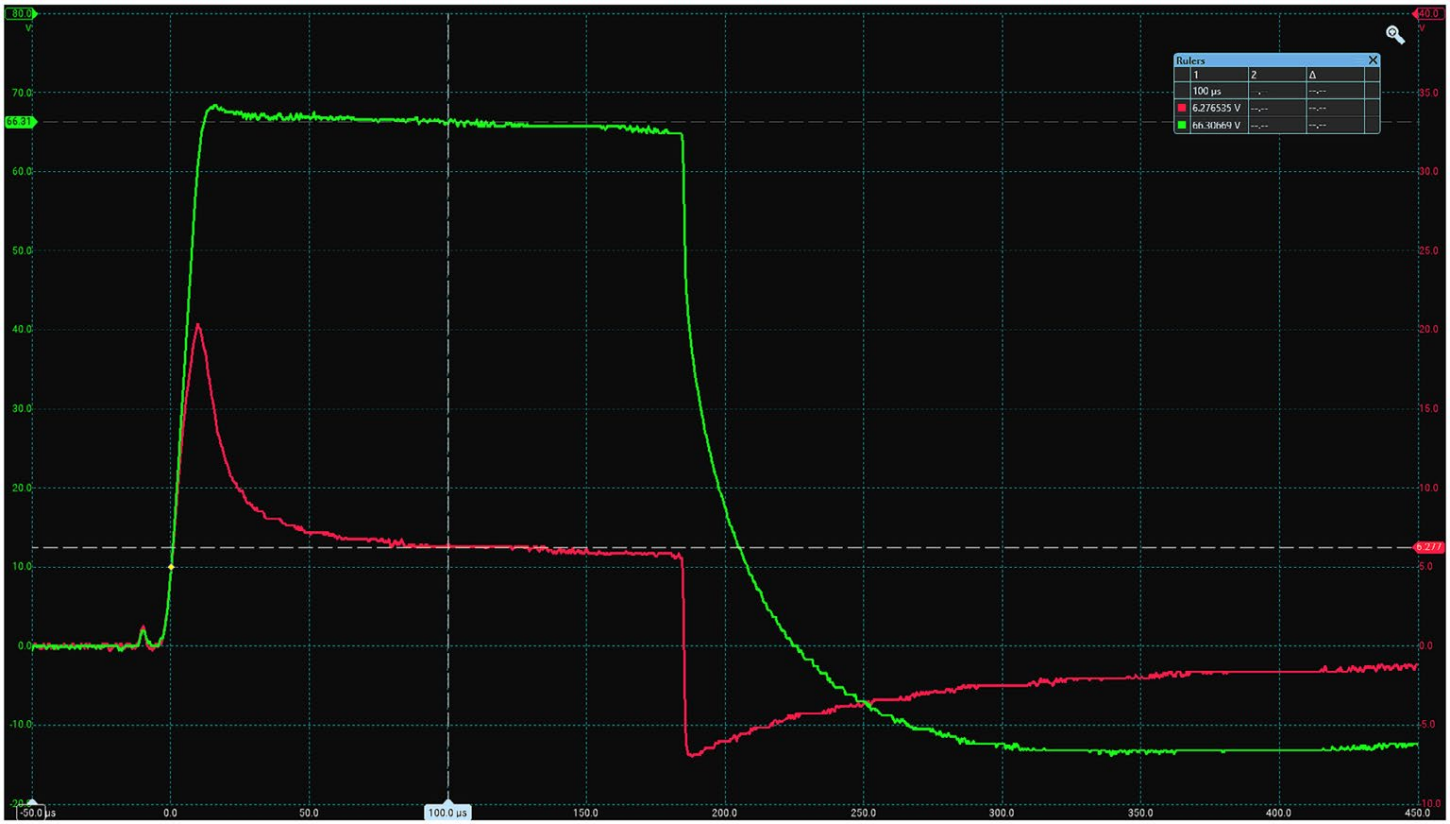
Oscilloscope trace showing the applied 180 μs pulse from the stimulator (green) and the total current (red). While the current trace shows an initial surge, this settled to a near constant value before the measurements were taken at 100 μs. [Color figure can be viewed at wileyonlinelibrary.com]

The finite difference model developed for this work assumed that the reactance element of the tissue impedance was minimal in this application and therefore only considered the resistance between nodes. During the laboratory tests, the current flow was measured 100 μs after the start of each stimulation pulse, and by this time the current had settled to a near constant value (Figure 8). An initial current surge (lasting approximately 20 μs) was observed, indicating that there was a small capacitive element in the system. However, the charge (the area under the current plot) applied by the stimulator is the most significant factor in this application [16], and so this brief surge would have minimal effect. This suggested that modeling the hydrogel, skin, and subcutaneous tissue as a resistor network, and therefore neglecting the reactive components, was a reasonable assumption to make in these circumstances.

While the range of resistivity values used for the hydrogel, skin, and subcutaneous tissues in the model were reasonably representative, other factors, such as the effects of temperature variation on hydrogel resistivity, were not considered. It was assumed that the resistance values were consistent within each layer of the model (subcutaneous tissue, skin, and hydrogel) and this would not allow for local variations in resistivity. For future work, it could also be beneficial to simulate the voltage distribution in a larger area of subcutaneous tissue rather than only the region directly adjacent to the electrode array.

## 4 Conclusions

This work demonstrated that a relatively simple finite difference model can be used to simulate stimulation distribution under an electrode array. The simulation outputs were found to correlate with subsequent laboratory test results.

Discontinuous patterned hydrogel was shown to improve inter‐electrode resistance on electrode arrays relative to continuous hydrogel. This difference was found to become of increasing significance as the resistivity of the hydrogel decreased over prolonged periods of application to the skin. The superior performance of the patterned hydrogel was supported by both the modeling and laboratory testing. Following this work, the patent application including the discontinuous patterned hydrogel has been published [17].

The SHAPES randomized control trial, which is currently underway, is utilizing patterned hydrogel (manufactured using mechanical cutting techniques) to prolong the life of the electrode arrays. The patterned hydrogel is being used for periods of up to 4 weeks before being replaced, without significant loss in the focus of the stimulation over the array. In comparison with continuous hydrogel, the frequency at which the electrode arrays need to be replaced is reduced. This simplifies the usage of array stimulation for the participants, while simultaneously reducing costs. Initial feedback from the clinical trial shows that this approach is comfortable for the participants as well as being practical for them to use in their home environment. This demonstrates the viability of the discontinuous patterned hydrogel approach.

## Author Contributions

All authors were co‐applicants for the NIHR grant funding. Jamie Healey proposed the patterned hydrogel concept and designed the electrode arrays. Avril McCarthy provided oversight and project management. Mark Reeves developed the simulation model, designed and implemented the patterned hydrogel and laboratory testing, carried out the data analysis, and drafted the manuscript. All authors contributed to the preparation and approval of the final manuscript.

## Conflicts of Interest

The authors declare no conflicts of interest.

## Supporting information


Data S1.
